# Exploring the Cellular Impact of Size-Segregated Cigarette Aerosols: Insights into Indoor Particulate Matter Toxicity and Potential Therapeutic Interventions

**DOI:** 10.1021/acs.chemrestox.4c00114

**Published:** 2024-06-13

**Authors:** Yu-Xin Shen, Pe-Shuen Lee, Chia C. Wang, Ming-Chu Teng, Jhih-Hong Huang, Hsiu-Fang Fan

**Affiliations:** † Institute of Medical Science and Technology, National Sun Yat-sen University, Kaohsiung 804, Taiwan; ‡ Department of Chemistry, National Sun Yat-sen University, Kaohsiung 804, Taiwan; § Aerosol Science Research Center, National Sun Yat-sen University, Kaohsiung 804, Taiwan

## Abstract

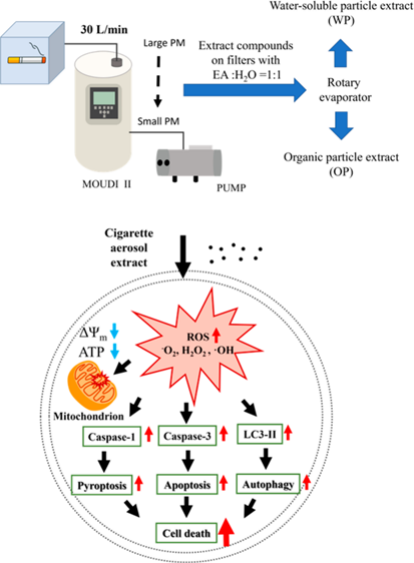

Exposure to anthropogenic aerosols has been associated with a variety of adverse health effects, increased morbidity, and premature death. Although cigarette smoke poses one of the most significant public health threats, the cellular toxicity of particulate matter contained in cigarette smoke has not been systematically interrogated in a size-segregated manner. In this study, we employed a refined particle size classification to collect cigarette aerosols, enabling a comprehensive assessment and comparison of the impacts exerted by cigarette aerosol extract (CAE) on SH-SY5Y, HEK293T, and A549 cells. Exposure to CAE reduced cell viability in a dose-dependent manner, with organic components having a greater impact and SH-SY5Y cells displaying lower tolerance compared to HEK293T and A549 cells. Moreover, CAE was found to cause increased oxidative stress, mitochondrial dysfunction, and increased levels of apoptosis, pyroptosis, and autophagy, leading to increased cell death. Furthermore, we found that rutin, a phytocompound with antioxidant potential, could reduce intracellular reactive oxygen species and protect against CAE-triggered cell death. These findings underscore the therapeutic potential of antioxidant drugs in mitigating the adverse effects of cigarette aerosol exposure for better public health outcomes.

## 1 Introduction

Exposure to toxic anthropogenic aerosols, suspended particulate matter (PM), has been linked to increased hospitalization and emergency room visits, mortality, and risk of premature death,^1−3^ as well as increased incidence and exacerbation of respiratory system-related diseases [such as asthma,^4^ chronic obstructive pulmonary disease (COPD),^5^ and cancers^6^, cardiovascular diseases (including arrhythmia, thrombosis, and atherosclerosis),^7^ brain-related diseases (such as Alzheimer’s disease and Parkinson’s disease),^8,9^ and kidney-related diseases (such as type-2 diabetes and chronic kidney disease),^10,11^ along with premature birth and fetal growth restriction.^12,13^ Different mechanisms, such as inertial impaction, gravitational sedimentation, Brownian diffusion, turbulent mixing, electrostatic precipitation, and interception, contribute to the deposition of PM.^14^ The deposition patterns of aerosols between 0.5 and 5.0 μm showed an increase in both overall and peripheral deposition as the particle size decreased.^15−17^ PM larger than 10 μm tends to settle in the nasopharyngeal area, PM between 5 and 10 μm can be found in the bronchial area, and those smaller than 2.5 μm reach the alveolar region. PM smaller than 0.1 μm is more likely to enter interstitial tissue.^18−20^ Smaller PM can potentially permeate through the epithelial barrier, entering the systemic circulation and affecting various organs.^17^ Moreover, it has been shown that 15 nm aerosolized quantum dots inhaled by mice can be found in the olfactory bulb and even neuron axons.^21^ Epidemiological studies have shown that fine PM is associated with increased mortality from cardiovascular and respiratory diseases, while coarse PM has weak or no significant correlation.^22−26^

Cigarette smoke poses one of the largest public health threats in the world, causing over 8 million deaths annually, with more than 7 million caused by direct cigarette use and approximately 1.2 million caused by exposure to secondhand smoke. Cigarette smoke is a complex system with adverse effects on the environment and human health.^27−29^ It contains both gas- and particulate-phase (referred to here as *aerosol*) components,^30^ including thousands of compounds,^31,32^ and more than 50 known carcinogens, such as polyaromatic hydrocarbons (PAHs) and tobacco-specific nitrosamines (TSNAs).^29,32^ According to the Centers for Disease Control and Prevention (CDC) of the United States, cigarette smoking or secondhand smoke exposure not only accounts for 80–90% of lung cancer deaths^33^ but is also linked to increased incidence of cardiovascular diseases and COPD.^34^ Secondhand smoke has been identified as a major source of indoor PM and a cause of lung cancer in nonsmokers, particularly in those exposed to heavy-smoking spouses.^35,36^ Passive smoking has been linked to an increased risk of various diseases and health problems, particularly in children, including asthma and cancer, and can lead to low birth weight in infants born to exposed mothers.^37−39^ The impact of both active and passive smoking on human health is significant.

Previous studies have collected total particulate matter, referred to here as *aerosol*, from cigarette smoke onto filter pads, extracted it with solvent, and named the extracts cigarette smoke condensate (CSC), referred to here as *cigarette aerosol extracts* (CAEs). The resulting gas phase from cigarette smoke was bubbled into fetal bovine serum (FBS)-free Dulbecco’s modified Eagle’s medium (DMEM) to prepare cigarette smoke extract (CSE).^40,41^ In vitro, genotoxicity assays are commonly used to investigate the biological activity of CSE and CSC.^42^ Although the effects of cigarette smoke on various cells have been explored, the extraction procedures and cell lines mentioned in recent studies vary,^43−51^ complicating comparisons. It has been proposed that smaller aerosols tend to deposit deeper into the respiratory tract system and thus potentially affect various organs more severely.^23−26,52^ However, the cytotoxicity of cigarette aerosols in cigarette smoke has never been systematically examined in a size-segregated manner. Furthermore, cigarette smoke can be classified into mainstream smoke, which mostly affects active smokers, and side-stream smoke, which mainly affects passive smokers.^53^ Most studies using cellular platforms have focused on investigating the influence of mainstream smoke,^40,41,43,45,46,48−51^ leaving the cellular effect of side-stream smoke relatively unexplored. To address these issues, this study investigates the effects of side-stream cigarette aerosols collected by size. Cigarette aerosol contains thousands of compounds,^31,32^ including more than 50 known carcinogens, such as polyaromatic hydrocarbons (PAHs) and TSNAs.^29,32^ Most studies prepare the CSE using aqueous buffer solution or DMEM, which leaves the influence of nonpolar compounds uninvestigated.^40,41,43−51^ To address this gap, our study focuses on preparing CAE according to their polarities. Exposure to cigarette smoke has been linked to an increased risk of various diseases and health problems.^54,55^ It has been proposed that PM can potentially permeate through the epithelial barrier, entering the systemic circulation and affecting various organs.^17^ Therefore, we examined the influence of the obtained CAE on three different cell lines, each originating from various tissues, to understand the broad spectrum of CAE’s potential toxicity. The SH-SY5Y cell line is a thrice-cloned subline of the neuroblastoma cell line SK-N-SH, established in 1970 from a metastatic bone tumor of a 4 year old cancer patient. This cell line is commonly used as a transfection host and in immunology, neuroscience, and toxicology research. Recently, it has been applied to investigate PM-induced cytotoxicity to mimic PM-induced neurotoxicity.^56^ A549 cells, isolated from the lung tissue of a 58 year old white male with lung cancer, have been extensively used in cancer, immuno-oncology, and toxicology research. Recently, this cell line has been employed to investigate PM-induced cytotoxicity, mimicking PM-induced lung toxicity.^57−59^ Similarly, 293T cells, epithelial-like cells isolated from the kidney of a patient, have been utilized to study PM-induced cytotoxicity as a model for in vitro kidney toxicity.^60^ In this study, we used SH-SY5Y cells to investigate CAE’s neurotoxic effects, A549 alveolar epithelial cells to assess the impact on lung tissue, and HEK293T kidney cells to explore the effects on kidney health. These cell lines were chosen to illustrate the CAE’s diverse impacts across critical body systems, highlighting the need for a comprehensive understanding of exposure to cigarette aerosols and the underlying mechanisms involved. Finally, we investigated the potential of rutin, a natural compound known for its antioxidative properties, in mitigating the adverse effects of CAE on each chosen cell line.

## 2 Materials and Methods

### 2.1 Cigarette Aerosol Collection and Preparation of CAE

**Figure 1:**
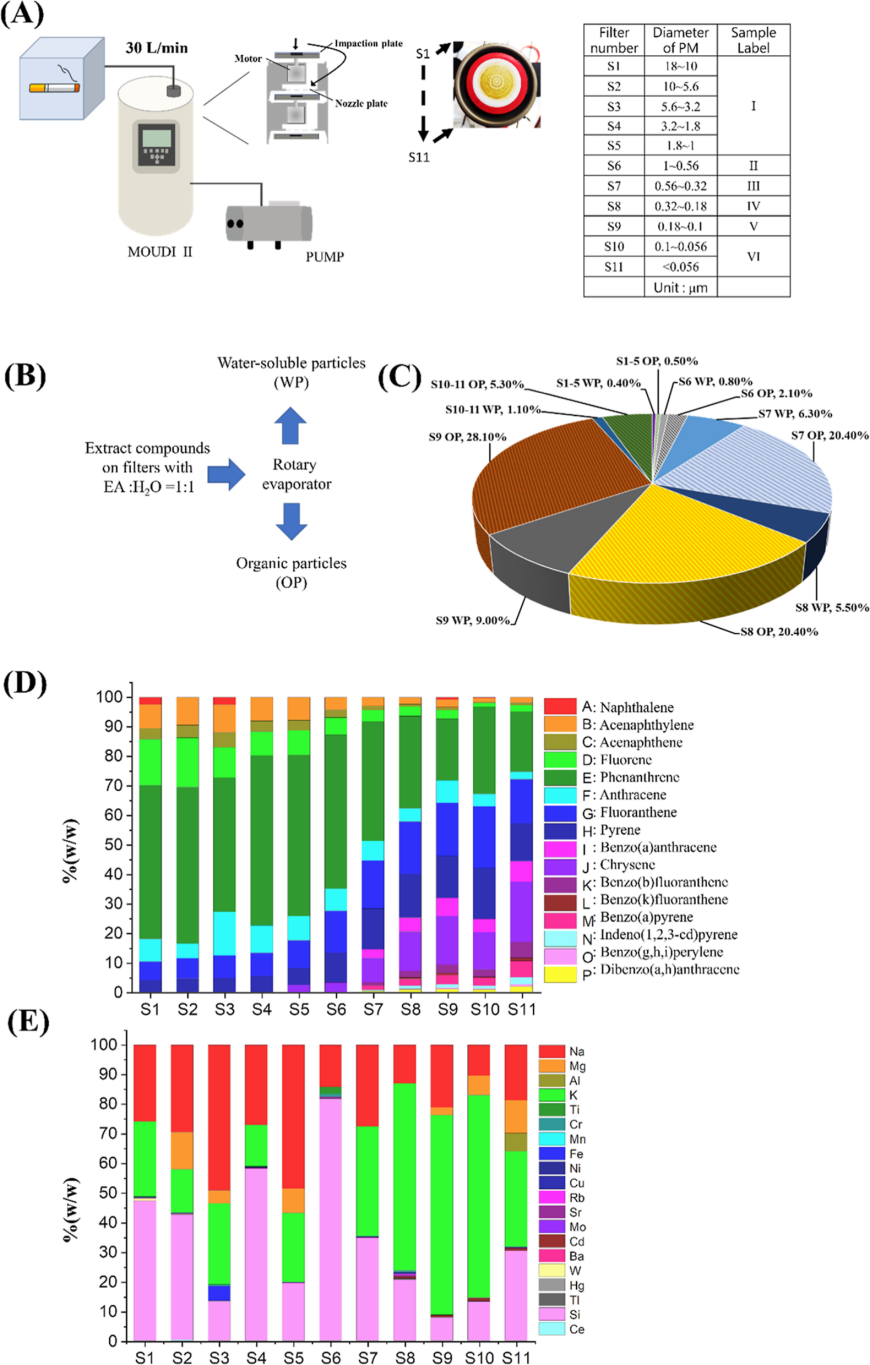
(A) Cigarette aerosol collection procedure and the classification table. (B) Extraction preparation procedure. (C) Mass distribution of CAE. (D) Relative mass distribution of 16 PAHs in cigarette aerosol collected. (E) Relative mass distribution of heavy metal components in cigarette aerosol collected.

The cigarette (5 mg tar and 0.4 mg nicotine/cigarette, Longlife Gentle 6, Taiwan) was combusted in a homemade smoking chamber. The combustion was maintained at room temperature (approximately 22 °C) and 1 atm pressure to closely mimic the formation of side-stream cigarette smoke under normal combustion conditions. Cigarette aerosol samples were collected on polytetrafluoroethylene filters (Finetech, M-PTFE047N010O) with the Micro-Orifice Uniform Deposit Impactor (model 120 MOUDI II Impactor, USA), according to different size ranges (S1: 18–10 μm, S2: 10–5.6 μm, S3: 5.6–3.2 μm, S4: 3.2–1.8 μm, S5: 1.8–1 μm, S6: 1–0.56 μm, S7: 0.56–0.32 μm, S8: 0.32–0.18 μm, S9: 0.18–0.10 μm, S10: 0.10–0.056 μm, and S11: <0.056 μm) with a pump flow rate of 30 L min^–1^ (GAST,1023–101Q-SG608X). The collection system, including the combustion chamber and MOUDI II Impactor, was disassembled and cleaned with detergent, an extra amount of water, followed by an ethanol rinse, and then evaporated in the hood. The connection tubes used in the collection system were also cleaned with ethanol and dried with nitrogen to prevent contamination. The collected filters were stored at −80 °C until extraction (Figure 1A).

For a size-dependent experiment, collected cigarette aerosol samples were further pooled according to the diameter of PM and labeled with fractions I–VI (listed in Figure 1A). The content mass ratio of PAHs bound in cigarette aerosols in each stage (S1–S11 mentioned in Figure 1A) was detected by using gas chromatography–mass–mass spectrometry (GC–MS–MS) (Figure 1D). The concentrations of main metal ions in each stage were measured by using inductively coupled plasma mass spectrometry (Figure 1E). The contents of the organic compounds in each stage were analyzed by using liquid chromatography–MS (LC–MS) (Figures S1 and S2). Detailed analysis procedures are provided in the supplementary methods.

For the preparation of CAE, the filters collected at different stages of MOUDI, loaded with cigarette aerosols of different diameters, were cut into small pieces (1 × 1 cm) and immersed into a mixture solution composed of ddH_2_O and ethyl acetate. The water-soluble components (WP) and organic components (OP) were extracted using a shaker for 20 min at ambient temperatures of 20–25 °C. The aqueous and ethyl acetate phases were evaporated to dryness at 40 °C using a rotary evaporator. The resultant dry powders were weighed, stored at −80 °C (Figure 1B), and reconstituted to a final concentration of 25 mg/mL in ddH_2_O and dimethyl sulfoxide (DMSO), respectively, for subsequent cytotoxicity testing and toxicological evaluations. Cytotoxicity assays on SH-SY5Y, HEK293T, and A549 cell lines confirmed that DMSO exhibited minimal impact on cell viability, with cell survival rates consistently above 90% under the tested conditions (Figure S3).

### 2.2 Cell Culture and Treatment

Human embryonic kidney (HEK293T) cells, human lung adenocarcinoma cells (A549), and human neuroblastoma (SH-SY5Y) cells were cultured in high-glucose DMEM (Gibco, 11965-084) supplemented with 1% penicillin/streptomycin (Gibco, 15140-122) and 10% (v/v) FBS (Corning, 35-010-CV). Cells were maintained in 10 cm culture dishes (α-plus, 16203-1SS) at 37 °C in a saturated humidity atmosphere containing 95% air and 5% CO_2_.

### 2.3 Cell Viability Assay

HEK293T cells, A549 cells, and SH-SY5Y cells were seeded at a density of 3.5 × 10^4^ cells/well in 96-well plates (α-plus, 116196-1SS) and treated with various concentrations of WP CAE (500, 700, 900, 1100, 1300, 1500, 1700, and 1900 μg/mL) and OP CAE (5, 10, 50, 75, 100, 150, and 200 μg/mL) for 24 h. Cell viability was assessed using the MTT (3-(4,5-dimethylthiazole-2-yl)-2,5-diphenyltetrazolium bromide) assay (Alfa Aesar, L11939).^61^ After 24 h of CAE treatment, the medium was removed, and cells were washed with phosphate buffer (137 mM NaCl, 2.7 mM KCl, 10 mM Na_2_HPO_4_, and 1.8 mM KH_2_PO_4_). Fresh DMEM containing MTT (0.5 mg/mL) was then added, and cells were incubated for 4 h at 37 °C in a humidified atmosphere of 95% air and 5% CO_2_. After that, the solution was removed, and cells were washed with phosphate buffer (137 mM NaCl, 2.7 mM KCl, 10 mM Na_2_HPO_4_, and 1.8 mM KH_2_PO_4_). The formazan formed was solubilized in 300 μL of DMSO (Cyrusbioscience, 101-67-68-5). Absorbance was measured at 570 nm using a microplate reader (SpectraMax i3, Molecular Devices). Statistical analysis of the difference groups was analyzed using the paired *t*-test.

### 2.4 Detection of Intracellular Reactive Oxygen Species (ROS) (H_2_O_2_) Generation

HEK293T cells, A549 cells, and SH-SY5Y cells were seeded at a density of 3.5 × 10^4^ cells/well in 96-well plates (α-plus, 116196-3SB). For measurement of intracellular hydrogen peroxide (H_2_O_2_), the OxiVision Green hydrogen peroxide sensor (AAT Bioquest, 11503)^62,63^ was preincubated with cells for 1 h before CAE treatment (OP: 55 μg/mL for SH-SY5Y and 200 μg/mL for HEK293T and A549) for an additional 80 min at 37 °C. Fluorescence intensity inside the cells was quantified using a microplate reader (Molecular Devices, SpectraMax iD3) with an excitation wavelength of 490 nm and an emission wavelength of 525 nm.

### 2.5 Detection of Mitochondrial ROS (•OH and O_2_^–^) Generation

HEK293T cells, A549 cells, and SH-SY5Y cells were seeded at a density of 3.5 × 10^4^ cells/well in 96-well plates (α-plus, 116196-3SB). To measure mitochondrial hydroxyl radical (•OH) generation, cells were first preincubated with 100 μL of 1.25 × MitoROS OH580 (AAT Bioquest)^63,64^ for 60 min. Then, cells were treated with CAE (OP: 55 μg/mL for SH-SY5Y and 200 μg/mL for HEK293T and A549) for an additional 4 h at 37 °C. Intracellular fluorescence intensity was quantified using a microplate reader (Molecular Devices, SpectraMax iD3) with excitation and emission wavelengths set at 540 and 590 nm, respectively. To assess mitochondrial superoxide (O_2_^–^) production, cells were treated with CAE (OP: 55 μg/mL for SH-SY5Y and 205 μg/mL for HEK293T and A549) for 4 h 37 °C. Then, 100 μL of 1.25 × MitoROS 580 (AAT Bioquest)^65,66^ was added for an additional 60 min at 37 °C. Fluorescence intensity was measured under the same conditions as above. Statistical significance between groups was determined using a paired *t*-test.

### 2.6 Mitochondrial Membrane Potential Assay

HEK293T cells, A549 cells, and SH-SY5Y cells were plated at a density of 3.5 × 10^4^ cells/well in 96-well plates (α-plus, 116196-3SB). For measurement of mitochondrial membrane potential (MMP), cells were preincubated with 100 μL of JC-1 (Abcam, ab113850)^67,68^ for 10 min. Then, cells were washed with 1X dilution buffer twice before treatment with CAE (OP: 55 μg/mL for SH-SY5Y and 200 μg/mL for HEK293T and A549) for an additional 150 min at 37 °C. Fluorescence was then measured using a microplate reader with dual excitation/emission settings for monomeric (475 nm/550 nm) and oligomeric (535 nm/590 nm) JC-1 forms.

### 2.7 ATP Assay

HEK293T cells, A549 cells, and SH-SY5Y cells were plated at a density of 3.5 × 10^4^ cells/well in 96-well plates (α-plus, 116196-3SB). For measurement of ATP level, cells were treated with CAE (OP: 55 μg/mL for SH-SY5Y and 200 μg/mL for HEK293T and A549) for 150 min at 37 °C. Then, 50 μL of detergent (supplied by Abcam, ab139484)^69,70^ was added and shaken for 5 min. Later, 50 μL of substrate solution (supplied by Abcam) was added and shaken for 5 min, followed by an additional 10 min of waiting in the dark. Luminescence intensity was measured with the microplate reader. Differences between experimental groups were analyzed using a paired *t*-test.

### 2.8 Capspase-1/-3 Activity Detection

Cells (1.0 × 10^6^) were treated with CAE (OP: 50, 75, and 100 μg/mL for SH-SY5Y and 100, 200, and 250 μg/mL for HEK293T and A549) for 24 h at 37 °C, followed by lysis using 50 μL of lysis buffer (provided by the manufacturer). Protein concentrations were determined using the Bradford method (Scientific Biotech Corp, BR01-500). The activities of caspase-1 and caspase-3 were detected using caspase activity assay kits, ab273268 (Abcam)^71^ and K106-25 (BioVision),^72,73^ respectively. Samples containing 150 μg of total protein were incubated with 50 μL of 2× reaction buffer (supplied by the manufacturer) and 5 μL of 4 mM YVAD-pNA/DEVD-pNA substrate (200 μM final concentration) for 2 h at 37 °C. Absorbance at 400 nm was recorded using a microplate reader (Molecular Devices, SpectraMax iD3). Percentage changes in caspase-1/-3 activity were calculated based on OD_400_ values. Statistical differences between groups were analyzed using a paired *t*-test.

### 2.8.1 Autophagy Activity Detection

HEK293T cells, A549 cells, and SH-SY5Y cells were plated at a density of 3.5 × 10^4^ cells/well in 96-well plates (α-plus, 116196-3SB). Autophagy activity was assessed using a fluorescence autophagy assay kit (Abcam, ab139484).^69,74^ Cells were treated with CAE (OP: 50 and 80 μg/mL for SH-SY5Y and 100 and 200 μg/mL for HEK293T and A549) for 4 h at 37 °C. Post-treatment, cells were incubated with the detection reagent (supplied by the vendor) for 30 min, followed by replacement with 100 μL of 1× assay buffer. Fluorescence intensity was measured using a microplate reader (Molecular Devices, SpectraMax iD3) with excitation at 480 nm and emission at 530 nm. Differences between groups were statistically analyzed using a paired *t*-test.

### 2.9 Immunofluorescence Analysis

HEK293T cells, A549 cells, and SH-SY5Y cells (6.0 × 10^4^ cells/well) were cultured on 22 mm coverslips (Marienfeld, AP-0111620) in a 12-well plate (α-plus, 16112) and treated with CAE (OP: 50 and 80 μg/mL for SH-SY5Y and 100 and 200 μg/mL for HEK293T and A549) for 4 h at 37 °C. Post-treatments, cells were fixed with 4% paraformaldehyde (ALFA, A11313) and permeabilized with 0.1% Triton ×-100 (Sigma-Aldrich, ×100) for 10 min. Next, cells were washed with phosphate-buffered saline (PBS) buffer three times and blocked with 5% bovine serum albumin (BioSHOP, ALB001) for 30 min. Later, cells were incubated with a 10 μg/mL antibody of LC3-B (Abcam, ab192890) at RT for 1 h. Then, cells were washed three times, followed by incubation with 4 μg/mL goat anti-rabbit IgG H&L (Alexa Fluor 488, Abcam, ab11150081) at RT for 1 h. After three-time washes with PBS, cells were incubated with 0.5 μg/mL 4′,6-diamidino-2-phenylindole (DAPI, Abcam, ab150081) to stain nuclei, followed by again three-time PBS wash. Then, cells were examined under home-built confocal fluorescence microscopy.

### 2.10 Rutin Treatment

For intracellular hydrogen peroxide (H_2_O_2_) measurement, the OxiVision Green hydrogen peroxide sensor (AAT Bioquest, 11503) was preincubated with cells at a density of 3.5 × 10^4^ cells/well in 96-well plates (α-plus, 116196-3SB) for 1 h before treatment of CAE (OP: 200 μg/mL for SH-SY5Y, 250 μg/mL for HEK293T and 300 μg/mL for A549) in the presence of various rutin concentrations (200, 250, and 300 μM, Acros Organics, AC132395000) for an additional 80 min at 37 °C. Fluorescence intensity was measured using a microplate reader (Molecular Devices, SpectraMax iD3) with an excitation wavelength of 490 nm and an emission wavelength of 525 nm. Cell viability was assessed via the MTT assay after a 24 h treatment with CAE and rutin. Post-treatment, cells were washed with phosphate buffer and incubated with MTT solution (0.5 mg/mL) for 4 h at 37 °C. The formazan product was dissolved in 100 μL of DMSO, and absorbance was measured at 570 nm using a microplate reader (SpectraMax i3, Molecular Devices). Statistical analysis was conducted using a paired *t*-test.

## 3 Results

### 3.1 Analysis of Chemical Characteristics of Cigarette Aerosols

According to a previous study, it has been reported that a typical aerosol particle size distribution for a 3R4F Kentucky Reference cigarette (Kentucky Tobacco Research & Development Center, Lexington, KY) measured with the low-flow impactor system exhibited a mass median aerodynamic diameter of 0.4 μm.^75^ Here, cigarettes used in this project were purchased from a local brand (Longlife Gentle 6, Taiwan), which is popular among smokers in Taiwan. After collecting the cigarette aerosols using MOUDI II, the aerosols were extracted and weighed separately. The mass-loaded populations on filters are shown in Figure 1A. It is observed that the most abundant cigarette aerosols were produced through traditional combustion range from 0.56 to 0.10 μm (Figure 1A), which is consistent with previously reported values.^75−79^ Since the mass-loaded values on S1–S5 were too low to be collected separately, all the compounds from S1–S5 were pooled together and labeled as fraction I. Compounds from S6, S7, S8, and S9 were labeled as fractions II, III, IV, and V, respectively. The same pooling procedure was applied to the compounds extracted from S10 and S11, which were labeled as fraction VI (Figure 1A). A higher population of organic-soluble compounds was obtained from the aerosols in comparison to water-soluble components (Figure 1B,C). Moreover, over 80% of the aerosols produced during the combustion process are smaller than 1 μm (Figure 1C). Indoors, tobacco smoke is considered one of the most important sources of PAHs.^80^ To date, 549 individual PAHs have been identified in tobacco smoke,^81^ with 12 of them classified as carcinogens by the IARC.^82^ During tobacco smoke combustion, PAHs are distributed between the gas and particulate phases.^81^ Despite their poor water solubility, PAHs and their metabolites have been found in blood, urine, and breast milk, indicating their absorption into the human body.^83,84^ Then, we evaluated the composition of PAHs in the collected cigarette aerosols. Sixteen standard PAHs were selected and analyzed. Based on our experimental results, regardless of the aerosol size, Phenanthrene was found to be the most abundant one among the 16 investigated PAHs (Figure 1D), consistent with a previous study.^85^ Moreover, the compositions of PAHs contained in cigarette aerosols smaller than 1 μm are more complex (Figure 1D). It has been reported that metal can enter the human body through cigarette smoking, indicating that metals in cigarette smoke can have harmful effects.^86^ Therefore, the IC analysis was used to investigate the metal compositions in the collected cigarette aerosols. The results revealed predominant concentrations of heavy metal components, notably, Na, K, and Si, accompanied by secondary levels of Mg, Al, and Fe, as well as trace quantities of various other heavy metals (Figure 1E). The LC–MS analysis results revealed that the collected cigarette aerosols in S1–S5 were insufficient, falling below the detection limit. Based on the mass analysis, the collected cigarette aerosols in S6–S11 exhibited nearly identical chemical compositions (Figures S1 and S2).

### 3.2 Effects of Size-Dependence Cigarette Aerosol Components on Cytotoxicity

**Figure 2:**
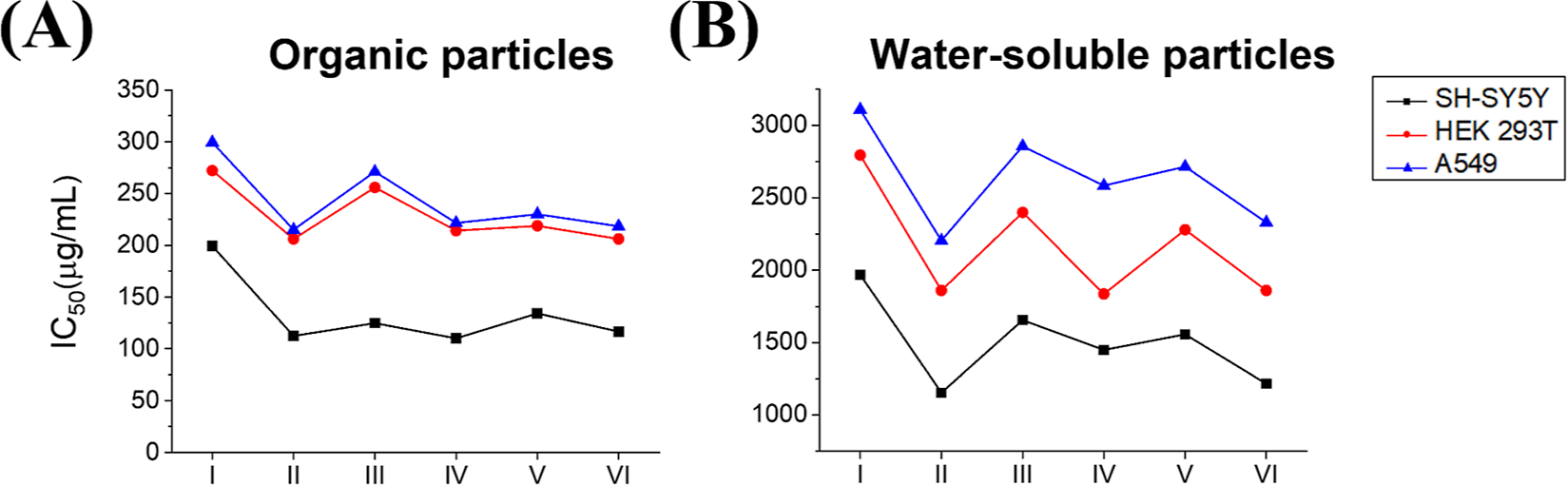
Distribution of IC_50_ values from three repeated tests of CAE effects on three cell lines, SH-SY5Y, HEK293T, and A549. The IC_50_ values of (A) OP extract and (B) WP extract. I–VI indicate the size of cigarette aerosol obtained with MOUDI listed in Figure 1.

Here, we treated three different cell lines, including human embryonic kidney (HEK293T) cells, human lung adenocarcinoma cells (A549), and human neuroblastoma (SH-SY5Y) cells, with various concentrations of WP CAE (500, 700, 900, 1100, 1300, 1500, 1700, and 1900 μg/mL) and OP CAE (5, 10, 50, 75, 100, 150, and 200 μg/mL) for 24 h. The MTT assay was used to investigate the cytotoxicity of the two extracts obtained from cigarette aerosol with different sizes. We observed a dose-dependent relationship between CAE concentration and cell viability, where cell viability gradually decreased with increasing CAE concentration (Figures S4 and S5) compared to the control (Figure S3). Comparing the IC_50_ values, which represent the concentration of the compound that causes a 50% reduction in cell viability or growth, it is evident that OP CAE exhibited higher toxicity than WP CAE. Furthermore, among the three investigated cell lines, the SH-SY5Y cell line was found to be more vulnerable. Interestingly, CAE from particles smaller than 1 μm showed greater cytotoxicity than those from larger particles (Figure 2). Because OP-CAE is more abundant and exhibits significantly greater cytotoxicity compared to WP-CAE, subsequent experiments were conducted using OP-CAE.

### 3.3 Effects of CAE on Oxidative Stress

**Figure 3:**
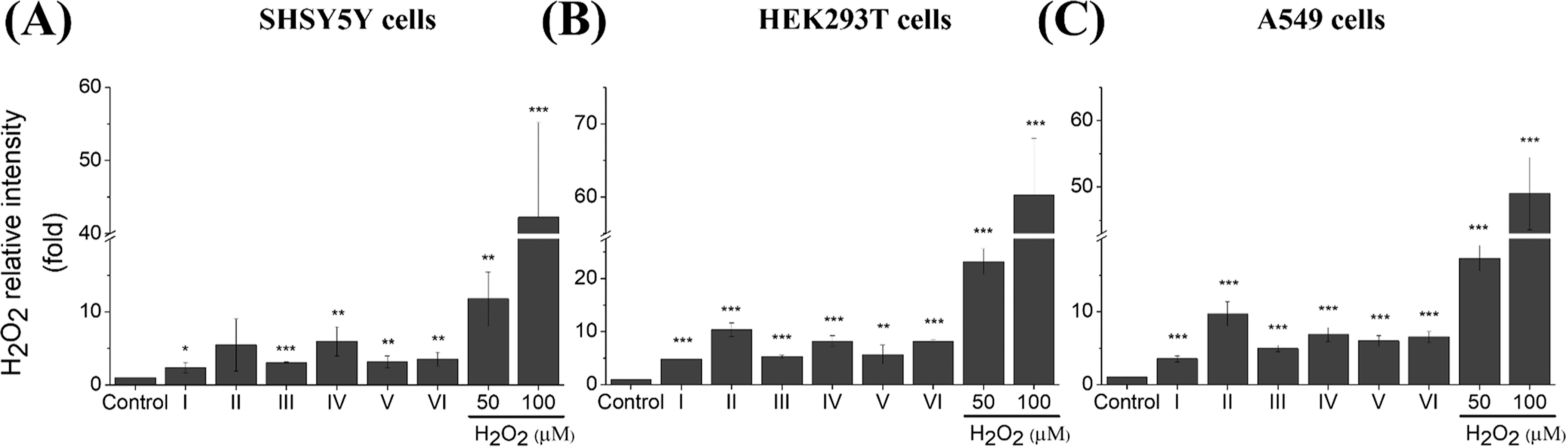
CAE promoted intracellular H_2_O_2_ generation in living cells, (A) SH-SY5Y, (B) HEK293T, and (C) A549. I–VI indicate the size of cigarette aerosol obtained with MOUDI listed in Figure 1. Data was calculated from three independent experiments (error bar, mean ± s.d.). *, **, and *** represent significant differences (* = *p* < 0.05), (** = *p* < 0.01), and (*** = *p* < 0.005) based on paired *t*-test in comparison to the control condition. All investigated conditions exhibit *p* < 0.005 for all three cell lines based on the analysis of variance (ANOVA) test as well.

ROS, such as superoxide (O_2_^–^), hydrogen peroxide (H_2_O_2_), and free radicals, can serve as signaling molecules.^87^ However, aberrant elevation in intracellular ROS levels or a decline in antioxidant capacity can lead to the accumulation of oxidative stress within cells. This, in turn, can cause damage to nucleic acids, proteins, and lipids, ultimately resulting in cell death,^88^ aging,^89^ and diseases.^90^ Recent studies have observed an elevation in ROS levels in cells following treatment with aerosol samples.^91−93^ Specifically, several studies have demonstrated that CSE disrupts mitochondrial function, leading to increased intracellular ROS production and inducing cell death.^93−96^ Therefore, we aimed to investigate whether the treatment of cells with CAE induces intracellular oxidative stress by measuring intracellular hydrogen peroxide (H_2_O_2_). The exposure of the three different cell lines to CAE resulted in a significant increase in the concentration of ROS compared to the control group (Figure 3). This observation suggests the exposure to CAE-induced oxidative stress in all the investigated cell lines. It is clearly observed that the CAE-induced ROS levels are anticorrelated with the IC_50_ values (Figures 2 and 3). Among the three investigated cell lines, all exhibited significant differences in ROS levels within fractions I–VI compared to the untreated condition, as validated by a paired *t*-test. CAE collected in fraction II (with cigarette aerosols ranging from 1.00 to 0.56 μm) and fraction IV (with cigarette aerosols ranging from 0.32 to 0.18 μm) induced higher ROS levels. However, there was no statistically significant difference in the induced ROS levels between fractions II and IV.

### 3.4 Effects of CAE on Biomarkers Related to Mitochondrial Function

**Figure 4:**
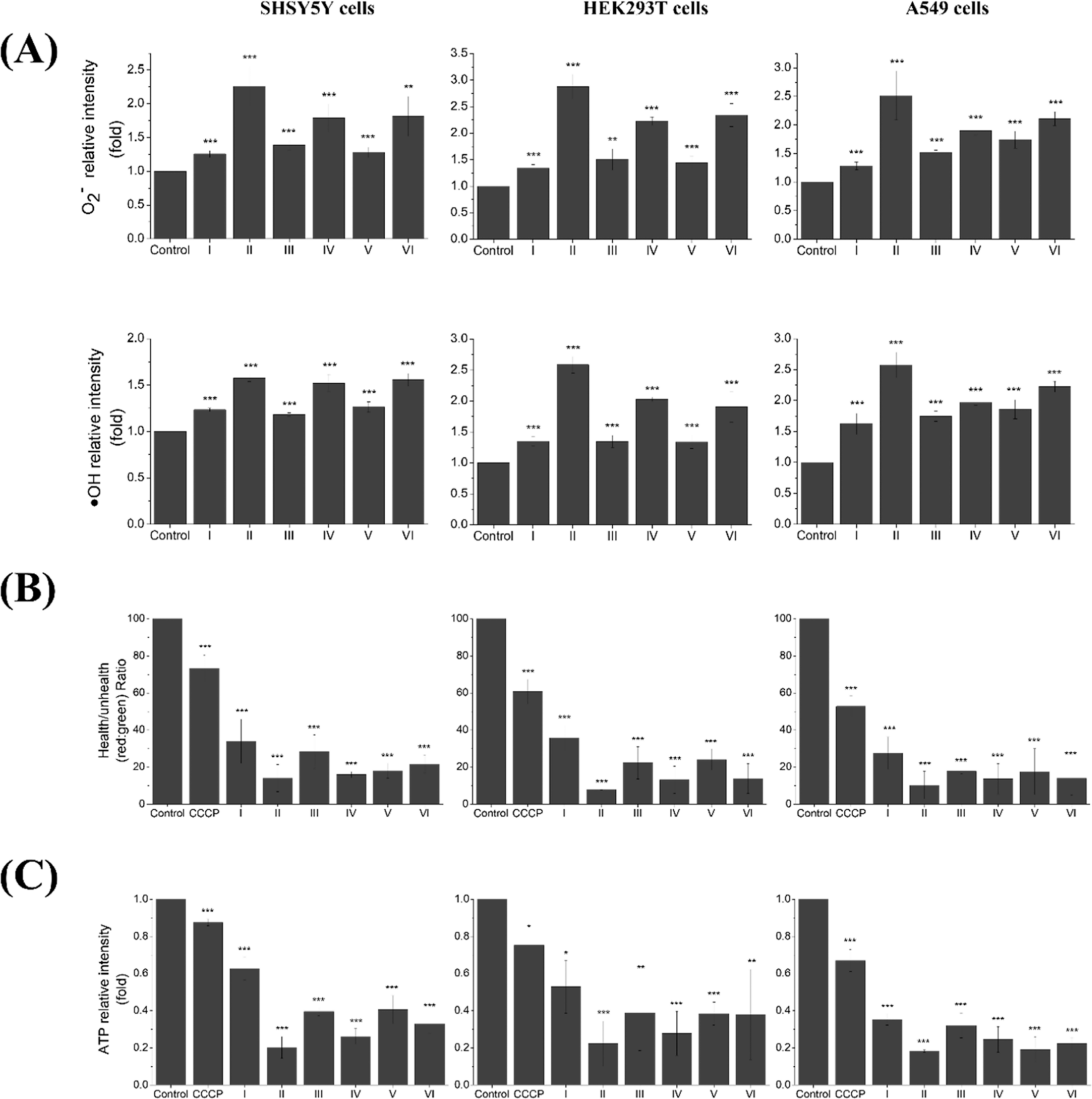
Influences of CAE on mitochondrial ROS generation, MMP polarization, and ATP generation in living cells, (A) SH-SY5Y (B) HEK293T, and (C) A549. I–VI indicate the size of cigarette aerosol obtained with MOUDI listed in Figure 1. Data was calculated from three independent experiments (error bar, mean ± s.d.). *, **, and *** represent significant differences (* = *p* < 0.05), (** = *p* < 0.01), and (*** = *p* < 0.005) based on paired *t*-test in comparison to the control condition. All investigated conditions exhibit *p* < 0.005 for all three cell lines based on the ANOVA test as well.

The exposure of mice to PM2.5 has been found to induce oxidative-redox imbalance and abnormal mitochondrial dynamics leading to mitochondrial damage.^97^ Similarly, it has been reported that exposure to PM2.5 resulted in a significant increase in mitochondrial fragmentation, reductions in mitochondrial mass and ATP levels, and a significant mitochondrial dysfunction in SH-SY5Y cells.^98^ Therefore, we would like to examine whether CAE could change mitochondrial ROS levels first. A549 cells were treated with different concentrations of OP CAE from the fraction III with cigarette aerosols ranging from 0.56 – 0.32 μm, causing a dose-dependent and time-dependent increase in mitochondrial ROS, including superoxide (O_2_^–^) and free radicals (Figure S6). Later, SH-SY5Y, HEK293T, and A549 cells treated with OP CAE from different fractions exhibited similar effects, also showing elevated levels of superoxide and free radicals in the mitochondria for all three cell lines (Figure 4A).

The MMP also serves as a crucial indicator of mitochondrial activity. Therefore, changes in mitochondrial membrane potential can be used to infer the normality of mitochondrial function.^99^ JC-1 (Abcam, ab113850) was used to measure the proportion of mitochondrial depolarization by monitoring the ratio of red fluorescence intensity to green fluorescence intensity. CCCP, a typical mitochondrial uncoupler, functions as a proton carrier that dissipates the transmembrane potential and depolarizes the mitochondria.^100^ Upon treatment with CCCP as a positive control, a decrease in red fluorescence is observed compared to the control group. In the case of A549 cells treated with OP CAE from the fraction III at different concentrations, a dose-dependent decrease in the ratio of red fluorescence intensity to green fluorescence intensity was observed (Figure S7). Similar trends were observed in all three different cell lines treated with OP CAE from different fractions, indicating that exposure to OP CAE significantly decreases the MMP (Figure 4B).

To monitor mitochondrial function, cells were treated with CAE (OP: 55 μg/mL for SH-SY5Y and 200 μg/mL for HEK293T and A549) for 150 min at 37 °C. The luminescent ATP detection assay (ab113849) was utilized to measure the ATP levels within the cells. In all three investigated cell lines, the ATP contents showed a significant decrease after treatment with OP CAE from different fractions compared to the control (Figure 4C). These findings demonstrate that exposure to OP CAE leads to significant increases in mitochondrial ROS, decreases in MMP, and impaired ATP generation capacity, indicating the occurrence of mitochondrial dysfunction following CAE treatment. From the above experiments, it is clearly observed that CAE-induced mitochondrial dysfunction is also anticorrelated with the IC_50_ values (Figures 2 and4), with fractions II and IV resulting in higher mitochondrial dysfunction.

### 3.5 CAE Induces the Programmed Cell Death

Programmed cell death encompasses three main forms: apoptosis, regulated necrosis, and autophagic cell death.^101,102^ Apoptosis involves extrinsic and intrinsic pathways, regulated by death receptors or mitochondria-controlled signaling.^103−105^ Pyroptosis is an inflammatory form of regulated necrosis mediated by inflammatory caspases (such as caspase-1/4/5/11). These caspases induce the formation of pores on the cell membrane, resulting in cellular swelling, rupture, and disruption of membrane integrity, leading to necrotic inflammation.^106−111^ Autophagy is a cellular process responsible for delivering damaged organelles, misfolded proteins, and other macromolecules to lysosomes for degradation and recycling.^112−114^ Moreover, autophagy is a survival mechanism but can lead to programmed cell death when dysregulated. In previous studies, it has been reported that CSE can induce pyroptosis,^115−117^ ferroptosis,^46,118,119^ autophagy, and apoptosis^48,120^ in different cell lines, suggesting the adverse effect of cigarette smoke.

Our experimental results indicate that exposure to CAE leads to significant cell death, accompanied by intracellular ROS accumulation, mitochondrial ROS accumulation, and pronounced mitochondrial dysfunctions related to ATP generation capability and MMP depolarization (Figures 2–4). Among all investigated CAE fractions (I–VI), CAE collected in fraction II and fraction IV showed greater cytotoxicity (Figures 2–4). However, due to the higher abundance of CAE obtained from cigarette aerosols deposited in fraction III and fraction V, subsequent experiments were conducted with CAE collected from fraction III to more easily investigate the adverse mechanisms after CAE treatment.

**Figure 5:**
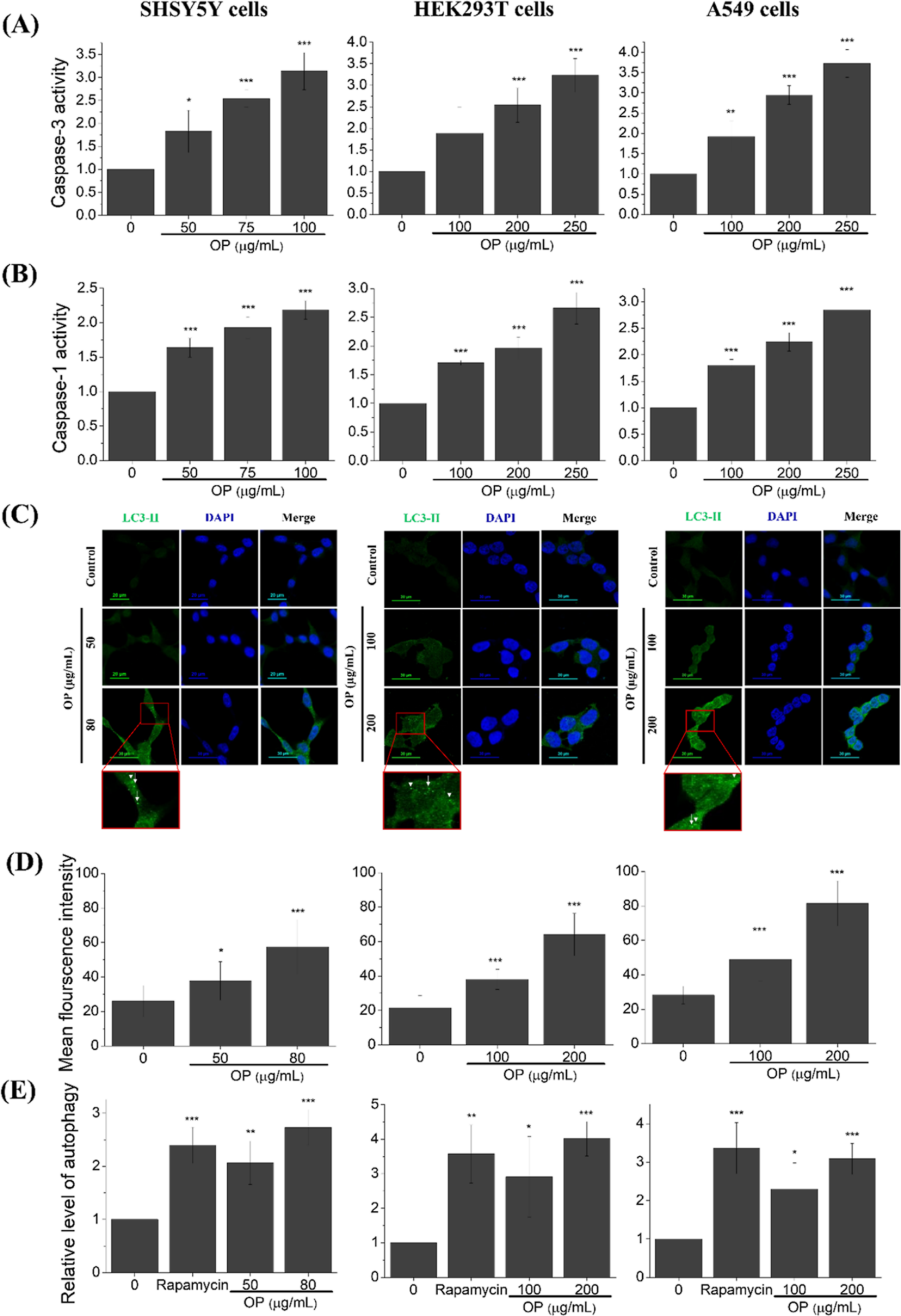
Influences of CAE on (A) caspase-3 and (B) caspase-1 activities, (C) representative immunofluorescence images of LC3-II, (D) quantified analysis of LC3-II images, and (E) autophagy activities in SH-SY5Y, HEK293T, and A549 cell lines. Data was calculated from three independent experiments (error bar, mean ± s.d.). *, **, and *** represent significant differences (* = *p* < 0.05), (** = *p* < 0.01), and (*** = *p* < 0.005) based on paired *t*-test in comparison to the control condition without CAE treatment.

Therefore, the next step is investigating whether cell death induced by CAE occurs through the apoptosis pathway. Caspase-3 activity in SH-SY5Y, HEK293T, and A549 cells treated with OP CAE from the fraction III showed a dose-dependent increase in all three cell lines, suggesting that CAE can induce cell death via the apoptosis pathway (Figure 5A). To confirm if cell death induced by CAE occurs through the pyroptosis pathway, caspase-1 activity was measured. The caspase-1 activity in SH-SY5Y, HEK293T, and A549 cells treated with OP CAE from the fraction III at different concentrations also exhibited a dose-dependent increase in all three cell lines, suggesting that CAE may also induce cell death through the pyroptosis pathway (Figure 5B). Last, to confirm if cell death induced by CAE occurs through the autophagy pathway, immunofluorescence staining of endogenous LC3-II and fluorescence-based autophagy assays was used to monitor autophagic activity. A dose-dependent increase in LC3-II fluorescence intensity and autophagy activity was observed (Figure 5C–E), suggesting that autophagy may act as a rapid response mechanism in the exposure of all three investigated cell lines to CAE. The experimental results demonstrate that OP CAE significantly triggers apoptosis, pyroptosis, and autophagy, ultimately leading to cell death.

### 3.6 CAE-Induced Cell Death Can Be Rescued by Potential ROS Scavenger

**Figure 6:**
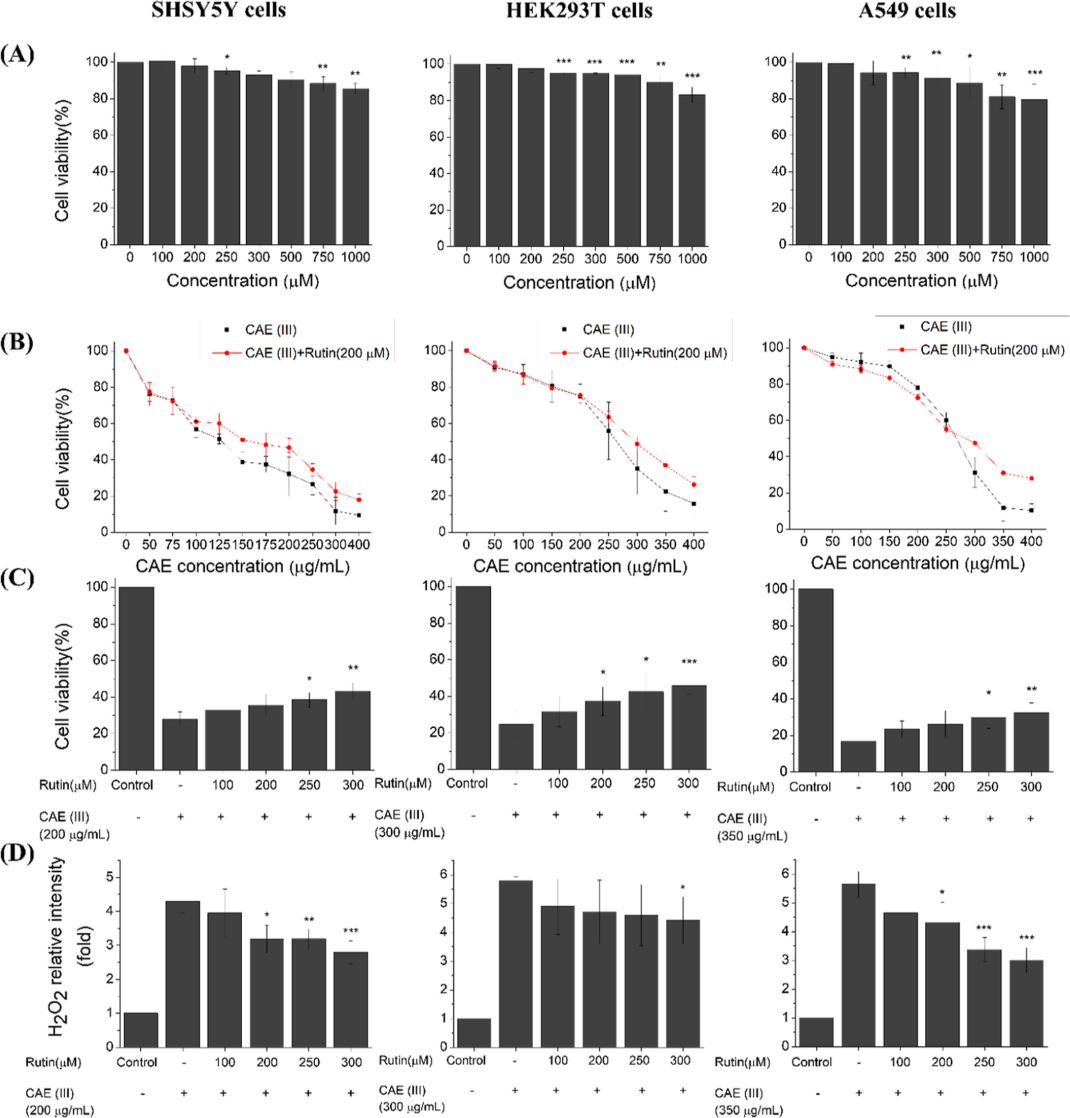
Rescue effects of rutin. (A). Cell viability of SH-SY5Y cells, HEK293T cells, and A549 cells after treatment with various concentrations of rutin for 24 h at 37 °C verified by the MTT assay. (B). Cell viability of SH-SY5Y cells, HEK293T cells, and A549 cells after treatment of OP CAE in the presence of 200 μM rutin for 24 h at 37 °C verified by the MTT assay. (C). Cell viability of SH-SY5Y cells, HEK293T cells, and A549 cells after treatment with OP CAE in the presence of various concentrations of rutin for 24 h at 37 °C verified by the MTT assay. (D) Intracellular H_2_O_2_ generation in SH-SY5Y, HEK293T, and A549 cells after treatment of OP CAE in the presence of various concentrations of rutin for 80 min at 37 °C. *, **, and *** represent significant differences (* = *p* < 0.05), (** = *p* < 0.01), and (*** = *p* < 0.005). *N* (repeat of experiment) is 3 for each condition based on paired *t*-test in comparison to the control condition. All investigated conditions exhibit *p* < 0.005 for all three cell lines based on the ANOVA test as well.

Rutin, a natural flavonoid found in plants, has shown pharmacological activities including anti-inflammatory,^121^ neuroprotective,^122^ cardioprotective,^123^ anticancer,^124^ and antiasthmatic effects.^125^ Our experimental results indicate that exposure to CAE results in the accumulation of intracellular ROS, leading to pronounced mitochondrial dysfunctions and significant cell death (Figures 2–4). Therefore, we evaluated rutin’s potential in mitigating CAE-induced cell damage. First, the cytotoxicity of rutin was assessed on SH-SY5Y, HEK293T, and A549 cells. Rutin exhibited minimal impact on cell viability, allowing for the selection of concentrations maintaining cell survival above 90%, with a maximum concentration of 300 μM for subsequent experiments (Figure 6A). For each cell line (SH-SY5Y, HEK293T, and A549), higher concentrations of CAE from the fraction III were chosen (200 μg/mL for SH-SY5Y, 300 μg/mL for HEK293T, and 350 μg/mL for A549). The cells were cocultured with different concentrations of rutin (100, 200, 250, and 300 μM) for 24 h, and the changes in cell viability were observed. The results showed that cell viability increased with higher concentrations of rutin (>200 μM), indicating its potential to mitigate the decrease in cell viability caused by CAE. It has been reported that rutin possesses powerful antioxidant effects. Here, we investigated whether rutin could alleviate oxidative stress-induced damage in living cells caused by CAE. After coculturing the cells with fraction III CAE (200 μg/mL for SH-SY5Y, 300 μg g/mL for HEK293T, and 350 μg/mL for A549) and different concentrations of rutin (100, 200, 250, and 300 μM) for 80 min, a decrease in intracellular H_2_O_2_ levels was observed with increasing concentrations of rutin, indicating that rutin can potentially reduce H_2_O_2_ production and mitigate the cell death induced by CAE in a concentration-dependent manner.

## 4 Discussion

When a cigarette is smoked, the smoke can be classified as mainstream or side stream smoke, both of which contribute significantly to indoor air pollution.^53^ Mainstream smoke particles range from 0.1 to 1.0 μm in size, while side-stream smoke particles are slightly smaller.^75,78,79^ The chemical constituents found in both mainstream and side-stream smoke possess the ability to permeate the lungs and be assimilated by cells. Smoking is a well-established source of exposure to hazardous chemicals, associated with a range of diseases. The objective of this study was to gather and examine the effects of these chemical constituents in cigarette aerosols based on their physical characteristics, such as particle sizes and solubilities in polar or nonpolar solvents.

From the collected CAE, we observed that the most abundant aerosols are in the range of 0.56–0.10 μm. CAE obtained from particles sized smaller than 1.00 μm exhibited higher cytotoxicity (Figures 1C and 2). The profiles of metals and PAHs appear to differ slightly between stages. Due to the limited amount of aerosols collected in S1–S5, it is difficult to prepare CAE separately, and the toxicity of specific aerosol sizes may be missed by grouping the filters in this manner. However, the mass load population indicates that most aerosols were around 1 μm in size for particles larger than 1.00 μm. Therefore, the properties of fraction I, including samples from S1–S5, may primarily derive from compounds collected in S5. Notably, the population of carcinogenic PAHs, including fluoranthene, pyrene, benzo(*a*)anthracene, chrysene, and especially benzo[*a*]pyrene (BaP)—known for their carcinogenic properties^126^—increases in smaller-sized aerosols (0.56–0.10 μm) (Figure 1D). The IARC lists BaP as a group-1 carcinogen, classifying it as the most potent carcinogen among the PAHs^127^ also dominantly found in smaller-sized aerosol. A comparison of the composition distribution of 16 investigated PAHs reveals that the composition complexity of CAE in the 0.56–0.10 μm range is greater than that from particles sized larger than1.00 μm. This observation suggests that smaller-sized cigarette aerosols produced during the combustion process may contain more complex and abundant chemicals. These factors may contribute to a lower IC_50_ and higher cytotoxicity as observed in this study. Our experiments revealed that OP CAE exhibited greater cytotoxicity than WP CAE, leading to significant cell death and lower IC_50_ values. This confirms that nonpolar components are more toxic compared to water-soluble components produced during the cigarette combustion process. Moreover, OP CAE is 3 to 4 times more abundant than WP CAE in all investigated fractions. Therefore, the influence of nonpolar chemical components on human exposure to cigarette aerosol is more severe. Among the three cell lines evaluated, SH-SY5Y demonstrated the greatest susceptibility to CAE, evidenced by a marked reduction in cell viability (Figure 2). In a related study, the cytotoxic effects of silver nanoparticles (AgNPs, 20 nm) were assessed across three cell lines (SH-SY5Y, A549, and D384), with SH-SY5Y cells showing the highest sensitivity.^128^ These findings suggest that SH-SY5Y cells may exhibit increased sensitivity to certain chemical exposures compared to A549 cells. Their neuronal properties might inherently make them more susceptible to certain toxic effects compared to lung epithelial (A549) or kidney (HEK293) cell lines, especially in the context of neurotoxic substances. Further research and targeted studies would be necessary to make definitive comparisons regarding cell line fragility under exposure to specific agents.

Previous studies have reported that CAE induces the production of ROS and mitochondrial ROS in bronchial epithelial cells.^43^ It has also been observed that CAE leads to a significant elevation in mitochondrial ROS and a decrease in ATP generation in HFL1 cells, resulting in a decline in MMP.^129^ Additionally, CAE has been found to reduce levels of mitofusin 2 (Mfn2), a protein involved in regulating mitochondrial fusion, thus disrupting the balance of mitochondrial fission/fusion and causing mitochondrial dysfunction.^130^ In our experiments, we observed a dose-dependent increase in intracellular ROS upon exposure to CAE. Lower IC_50_ values corresponded to higher ROS production, establishing a positive correlation between ROS generation and CAE-induced cytotoxicity (Figure 3). Moreover, CAE treatment led to a time-dependent and dose-dependent elevation of mitochondrial ROS, resulting in severe mitochondrial dysfunction (Figure 4). These findings corroborate the harmful effects of CAE on cellular and mitochondrial health, aligning with prior research.

It has been pointed out that the exposure of mice to cigarette smoke resulted in increased levels of ROS in lung tissues, along with elevated levels of cleaved caspase-3 protein compared to the control group. These observations indicate the activation of apoptosis.^131^ In another study, exposure to CSE was observed to enhance the activity of NLRP3 and caspase-1, leading to the release of interleukin-1β (IL-1β) and interleukin-18 (IL-18). These results suggest that cigarette aerosols induce inflammation and cellular pyroptosis through the ROS/NLRP3/caspase-1 pathway.^50^ Moreover, when BEAS-2B cells were treated with CSE, an increase in the levels of LC3B–II was observed, indicating the induction of autophagy.^51^ In our experiments, we quantified the activities of caspase-1, caspase-3, and LC3-II, which revealed a significant induction of apoptosis, pyroptosis, and autophagy-mediated cell death following exposure to CSE in SH-SY5Y, A549, and HEK293T cell lines. These results confirm that CAE collected in our experimental condition can trigger cell death by activating apoptosis, pyroptosis, and autophagy pathways in the three investigated cell lines, consistent with previous studies (Figure 5).

Free radicals are associated with the development of various chronic diseases.^132−134^ They can be produced in both mainstream and sidestream cigarette smoke,^135,136^ affecting both smokers and nonsmokers. Previous studies have demonstrated that active smokers are exposed to higher levels of free radicals, with increased ROS in their lungs and elevated peripheral white blood cell counts.^137,138^ These findings support the theory that free radicals contribute to the harmful effects of cigarette smoke. Rutin, a glycoside formed between the flavonol quercetin and the disaccharide rutinose, is believed to function as an antioxidant, a free radical scavenger, and an iron chelator.^139−145^ Upon hydrolysis, quercetin, which contains adjacent hydroxyl groups on the same benzene ring, is released and is especially prone to autoxidation, a process critical for the biological activity and stability of certain foods.^146,147^ In this study, rutin was found to mitigate oxidative stress-induced damage in living cells caused by CAE, with a slight improvement in cell viability observed following rutin treatment. This research highlights rutin’s potential role in protecting SH-SY5Y cells against CAE-induced cytotoxicity, which may be attributed to its ability to suppress oxidative stress within the cells. Previous studies have suggested that rutin might suppress ROS levels by regulating the JNK/TNF/P38 MAPK pathway.^148−150^ Further experiments should be conducted to confirm whether the potential role of rutin in protecting cells against CAE-induced cytotoxicity is also mediated through the regulation of the JNK/TNF/P38 MAPK pathway. Moreover, previous studies have explored the neuroprotective potential of rutin in SH-SY5Y cells exposed to neurotoxins such as 1-methyl-4-phenylpyridinium (MPP^+^) and rotenone.^148,151^ These studies have shown that rutin, at concentrations ranging from approximately 1–100 μM, exhibits therapeutic potential for treating neurotoxicity associated with oxidative stress. The concentration of rutin used in above-mentioned studies is lower than what is used here (∼100–300 μM). The inconsistency may arise from the different neurotoxins used (MPP^+^ and rotenone vs CAE). Moreover, rutin in a previous study was extracted from *Dendropanax morbifera* Leveille, which may contain additional bioactive compounds with neuroprotective properties.^148^ We demonstrate that this cellular platform can be adapted to screen potential candidates that alleviate CAE-induced cell death as a therapeutic strategy against toxic aerosol-induced oxidative stress. To achieve a better protective effect, the autoxidation behavior of rutin might be enhanced by the presence of transition-metal ions such as Fe (II) and Cu (II),^152^ which can be investigated with further experiments. Compared to other plant-derived bioactive compounds like curcumin, epigallocatechin-3-gallate (EGCG), and crocetin, known for their excellent antioxidant capabilities,^153−156^ rutin exhibits lower cytotoxicity, as indicated by its higher IC_50_ value.^157−159^ Rutin’s lower cytotoxicity compared to other plant-derived bioactive compounds is an advantage as it suggests that rutin may have safer oral dosage applications than these other compounds.

**Figure 7:**
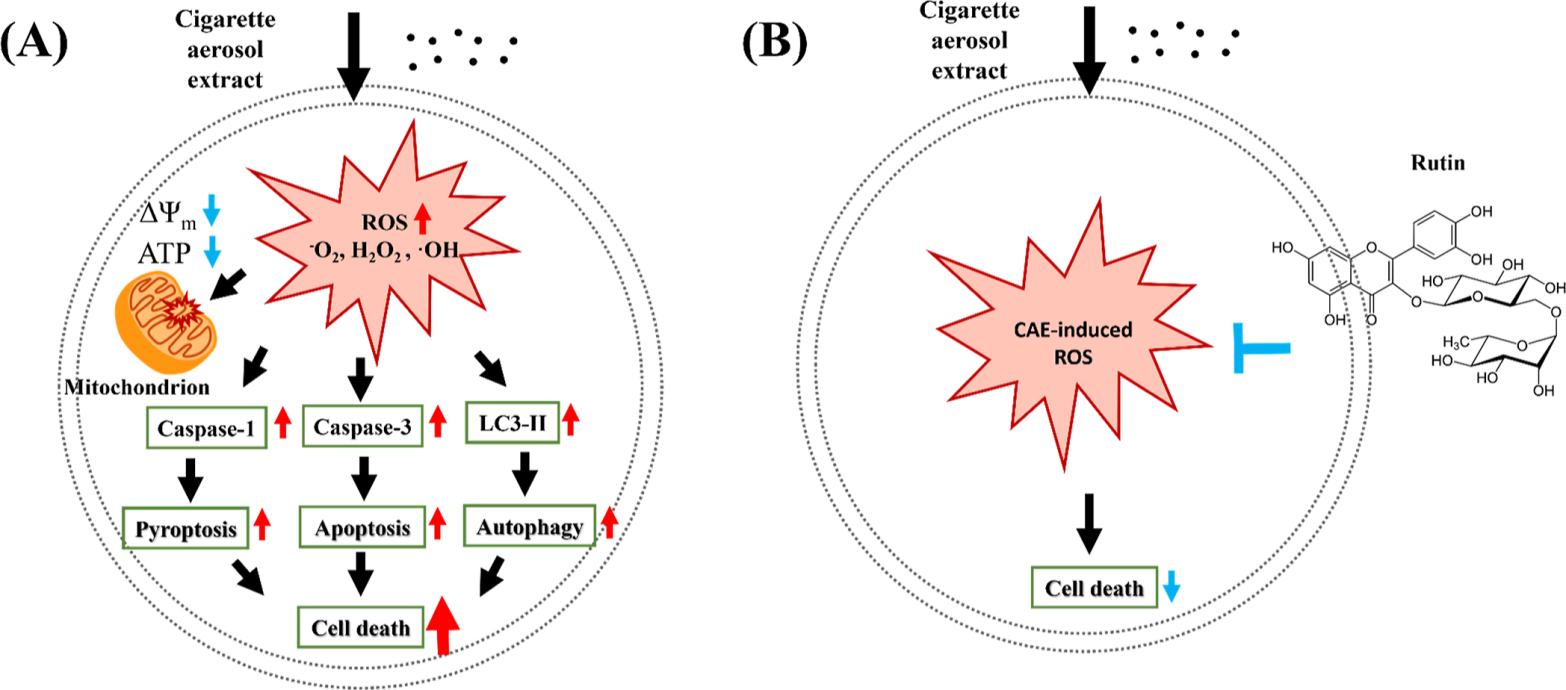
(A). Summary mechanism of CAE-induced cell death and (B) rescue effect of rutin treatment in living cells.

In summary, we systematically analyze the influence of size-segregated and polarity-defined CAE on three different cell lines. Here, we provide compelling evidence demonstrating that CAE (both WP and OP) can induce intracellular ROS accumulation, impair mitochondrial function, and trigger cell death via apoptotic, pyroptotic, and autophagic pathways in SHSY5Y, A549, and HEK293T cell lines. Furthermore, our work reveals the protective effects of rutin, a potent ROS scavenger, against CAE-induced cytotoxicity by attenuating intracellular ROS levels (Figure 7). These findings contribute to a deeper understanding of the cellular impact of cigarette aerosols and underscore the importance of protective strategies against cigarette smoke-induced cellular damage. Collectively, our findings unveil the intricate mechanisms underlying CAE-induced cell death, highlighting potential strategies for mitigating the adverse effects of cigarette smoke exposure.

## Supporting Information Available

LC–MS analysis results, DMSO influence on living cells verified by MTT assay, size-dependent OP and WP cigarette aerosol extraction influence on living cells verified by MTT assay, time-dependent and dose-dependent mitochondrial ROS in A549 cells treated with OP CAE, and the influence of cigarette aerosol OP extraction from fraction III on mitochondrial potential verified by the JC-1 assay (PDF)

## Abbreviations

CAE: cigarette aerosol extract
COPD: chronic obstructive pulmonary disease
MMAD: mass median aerodynamic diameter
MMP: mitochondrial membrane potential
OP: organic particle extract
PAHs: polycyclic aromatic hydrocarbons
PM: particulate matter
ROS: reactive oxygen species
WP: water-soluble particle extract (WP)
