# Supplementary material for: Exploring the Cellular Impact of Size-Segregated Cigarette Aerosols: Insights into Indoor Particulate Matter Toxicity and Potential Therapeutic Interventions
Source: Chem Res Toxicol. 2024 Jun 13;37(7):1171–86. doi: 10.1021/acs.chemrestox.4c00114 (PMC11256904; doi:10.1021/acs.chemrestox.4c00114)
Supplement: Supplementary file 1 — tx4c00114_si_001.pdf [file tx4c00114_si_001.pdf]

Supporting information

Exploring the Cellular Impact of Size-Segregated Cigarette Aerosols: Insights into Indoor Particulate Matter Toxicity and Potential Therapeutic Interventions

Yu-Xin Shen[1 2.3+], Pe-Shuen Lee[1 2.3+], Ming-Chu Teng[1 2.3], Jhih-Hong Huang[2.3], Chia C. Wang[2.3*], Hsiu-Fang Fan[1 2.3*]

[1] Institute of Medical Science and Technology, National Sun Yat-sen University, Kaohsiung, 804, Taiwan

[2] Department of Chemistry, National Sun Yat-sen University, Kaohsiung, 804, Taiwan

[3] Aerosol Science Research Center, National Sun Yat-sen University, Kaohsiung, 804, Taiwan

[4] Department of Life Sciences and Institute of Genome Sciences, National Yang-Ming University, Taipei, 112, Taiwan

***** Correspondence can be sent to***

CCW(@nsysu.edu.tw) and HFF (bendyfan@imst.nsysu.edu.tw)

To be submitted to Chemical Research in Toxicology

+ These authors have equal contributions to this work

Supplementary method:

For GC-MS/MS analysis, filters undergo initial microwave-assisted solvent extraction using hexane, acetone, and dichloromethane. The resulting extracts are then concentrated and processed through a multi-step cleanup using silica gel and alumina columns to isolate and purify various pollutants, including PBDEs, PAHs, NPAHs, and OPEs. These purified extracts are analyzed with an Agilent 7890A GC system coupled with an Agilent 7000D mass spectrometer, employing electron impact ionization.

For ICP-MS, samples are digested using either concentrated nitric acid or a mixture of nitric and hydrochloric acids, with microwave heating facilitating the extraction and digestion of elements from the solution. After cooling, the solution is filtered and diluted to the appropriate volume and analyzed using an Agilent 7900 Inductively Coupled Plasma Mass Spectrometry (ICP-MS).

The sample preparation for LC-MS analysis involves placing the filter paper in a 10 mL sample vial, adding 2 mL of methanol, and subjecting it to ultrasonication for 30 minutes, followed by a 5-minute rest. Next, 1 mL of the supernatant is extracted and centrifuged at 14,000 rpm for high-speed separation. Subsequently, 200 µL of the upper layer is collected for further analysis. LC-MS analysis was conducted using an Agilent ZORBAX Eclipse XDB-C18 column (4.6 mm × 250 mm, 5 µm, 40°C). Mobile phases consisted of 0.02% formic acid (A) and methanol (B), with a flow rate of 500 µL/min. An electrospray ionization (ESI) source-equipped tandem mass spectrometer detected ions in both positive and negative modes.

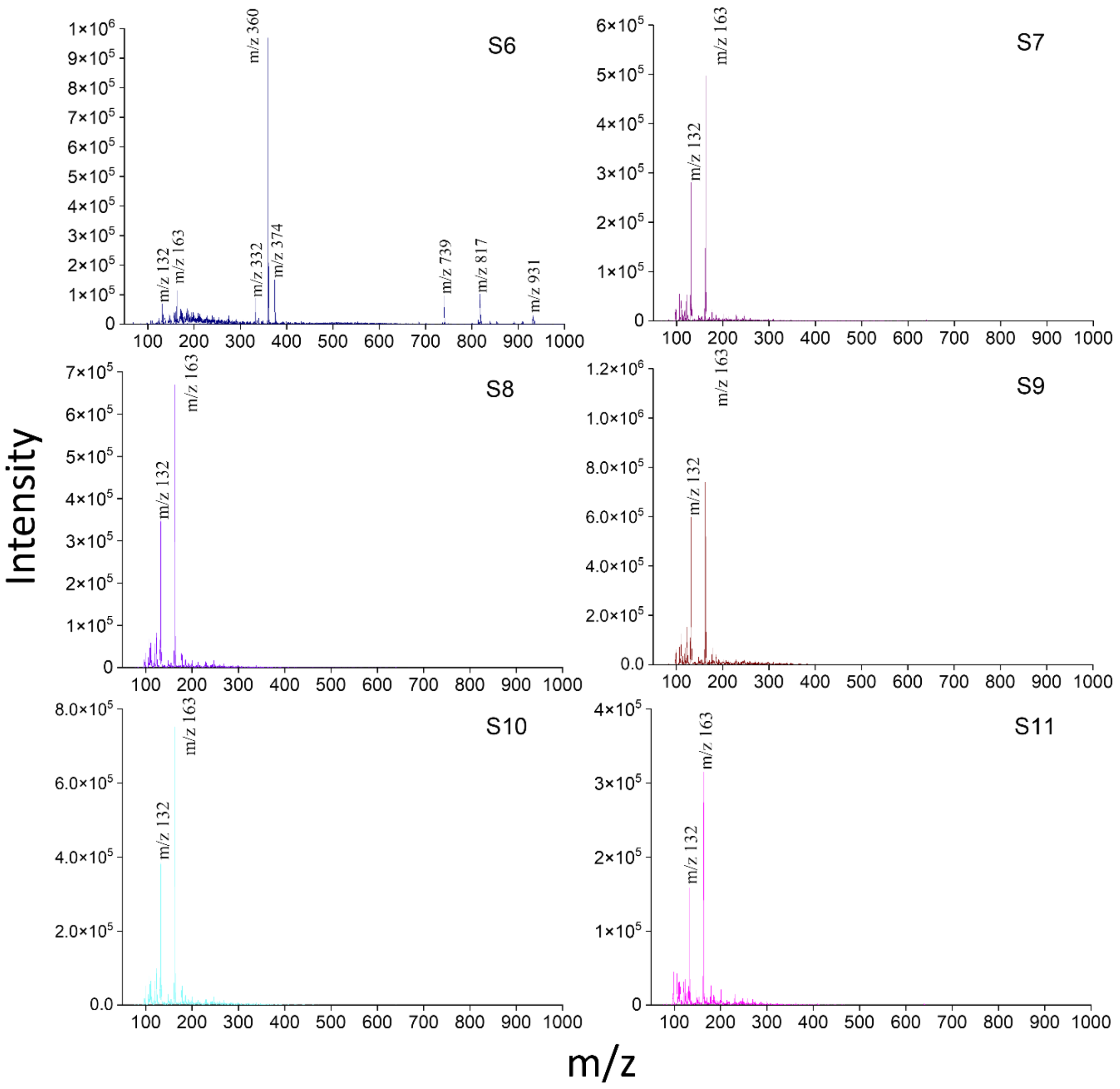

Figure S1 Liquid chromatography-coupled mass spectra in positive scan mode for cigarette smoke collected from stage 6 to stage 1. The intensity (cps, counts per second) is depicted on the Y-axis, while m/z is plotted on the X-axis. Compounds collected in stage 1 to stage 5 fall below the detection limit.

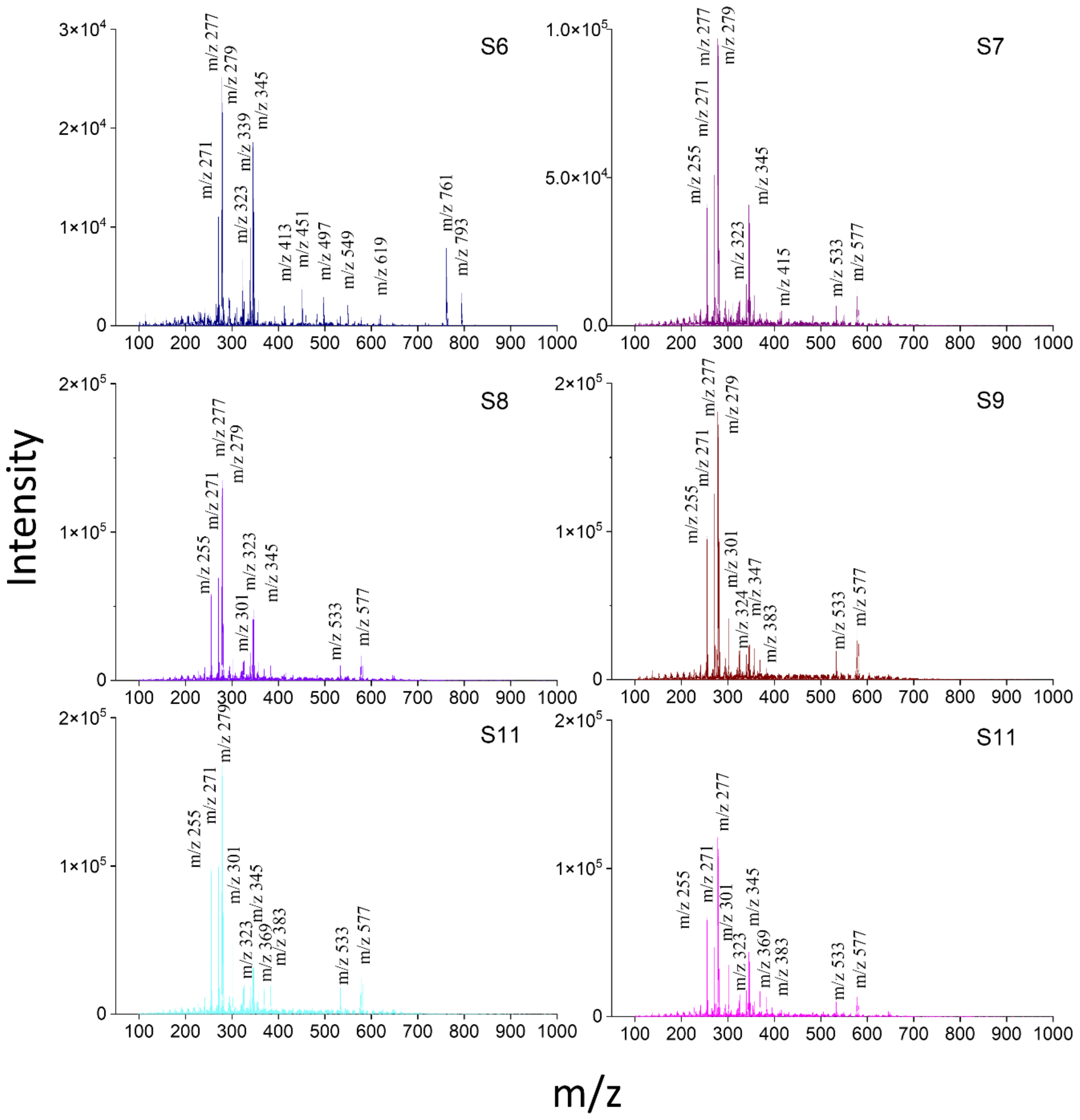

Figure S2 Liquid chromatography-coupled mass spectra in negative scan mode for cigarette smoke collected from stage 6 to stage 1. The intensity (cps, counts per second) is depicted on the Y-axis, while m/z is plotted on the X-axis. Compounds collected in stage 1 to stage 5 fall below the detection limit.

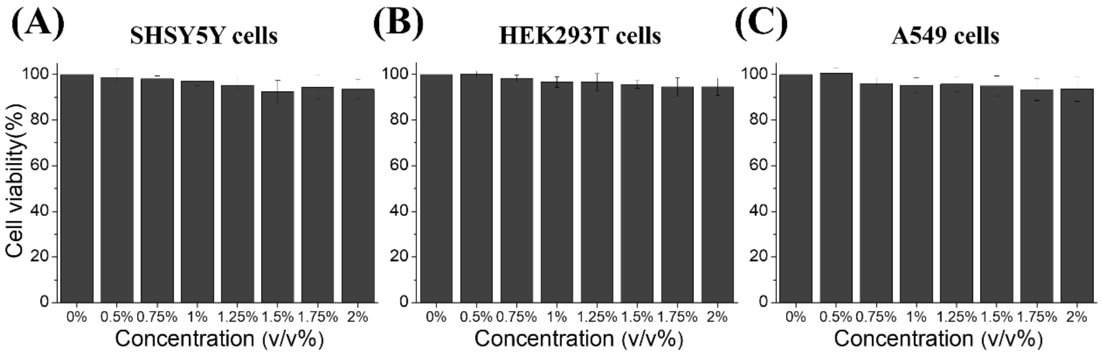

Figure S3 *DMSO influence on living cells verified by MTT assay.* Cell viability of (A) SH-SY5Y cells, (B) HEK293T cells, and (C) A549 cells after treatment with various concentrations of DMSO for 24 hours at 37°C verified by MTT assay. N (repeat of experiment) is 3 for each condition.

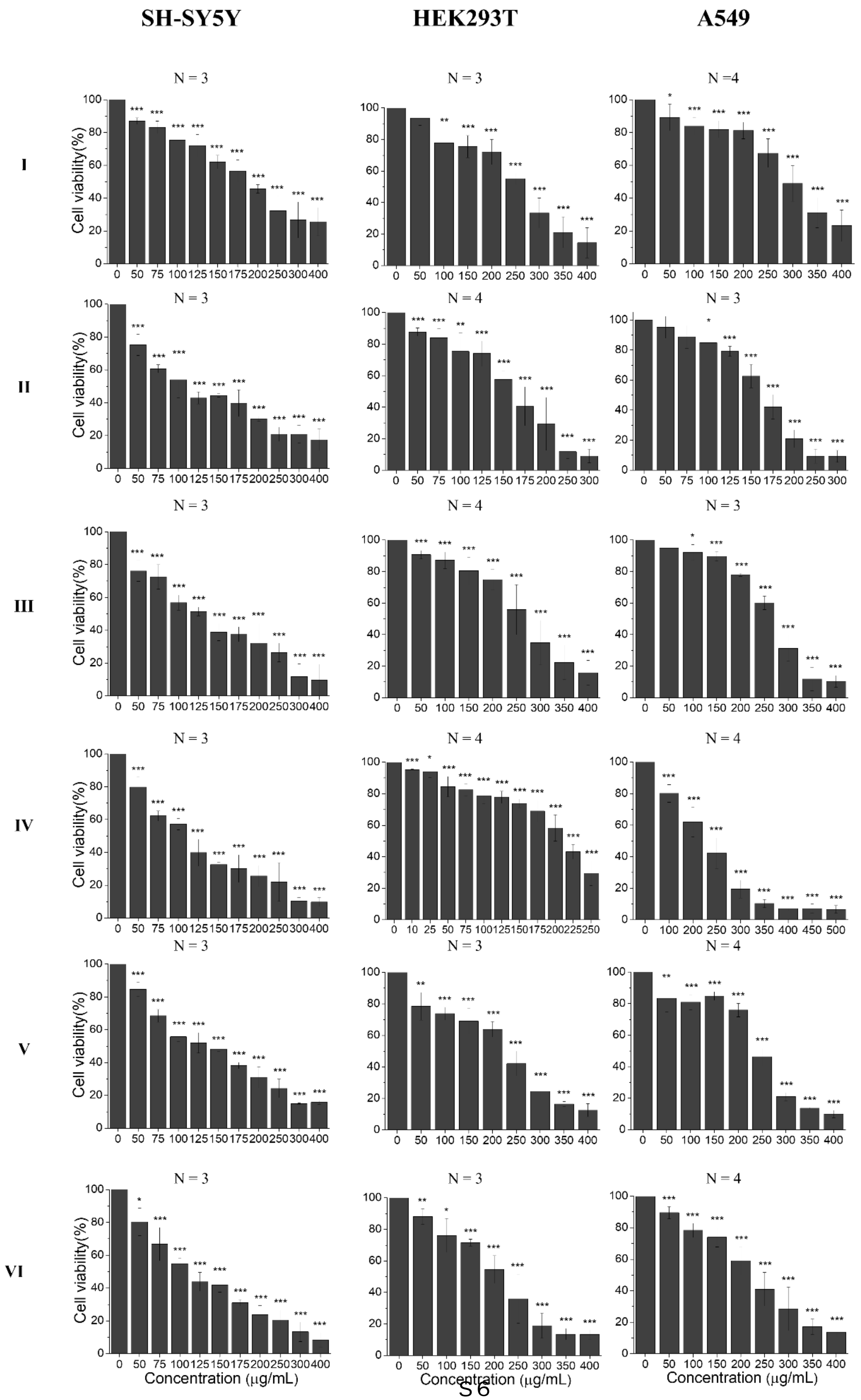

SH-SY5Y
HEK293T
A549
I
II
III
IV
V
VI
N = 3
N =4
N = 4
Cell viability(%)
Concentration (μg/mL)

Figure S4 *Size-dependent OP CAE influence on living cells verified by MTT assay.* Dose response curve of *CAE* in SH-SY5Y, HEK293T, and A549 cell lines using the MTT assays. *, **, and *** represent significant differences (* = $p < 0.05$), (** = $p < 0.01$) and (*** = $p < 0.005$). The numbers (N) indicate the number of experiments conducted in each condition. I~VI indicates the size of cigarette aerosol obtained with MOUDI listed in Fig. 1.

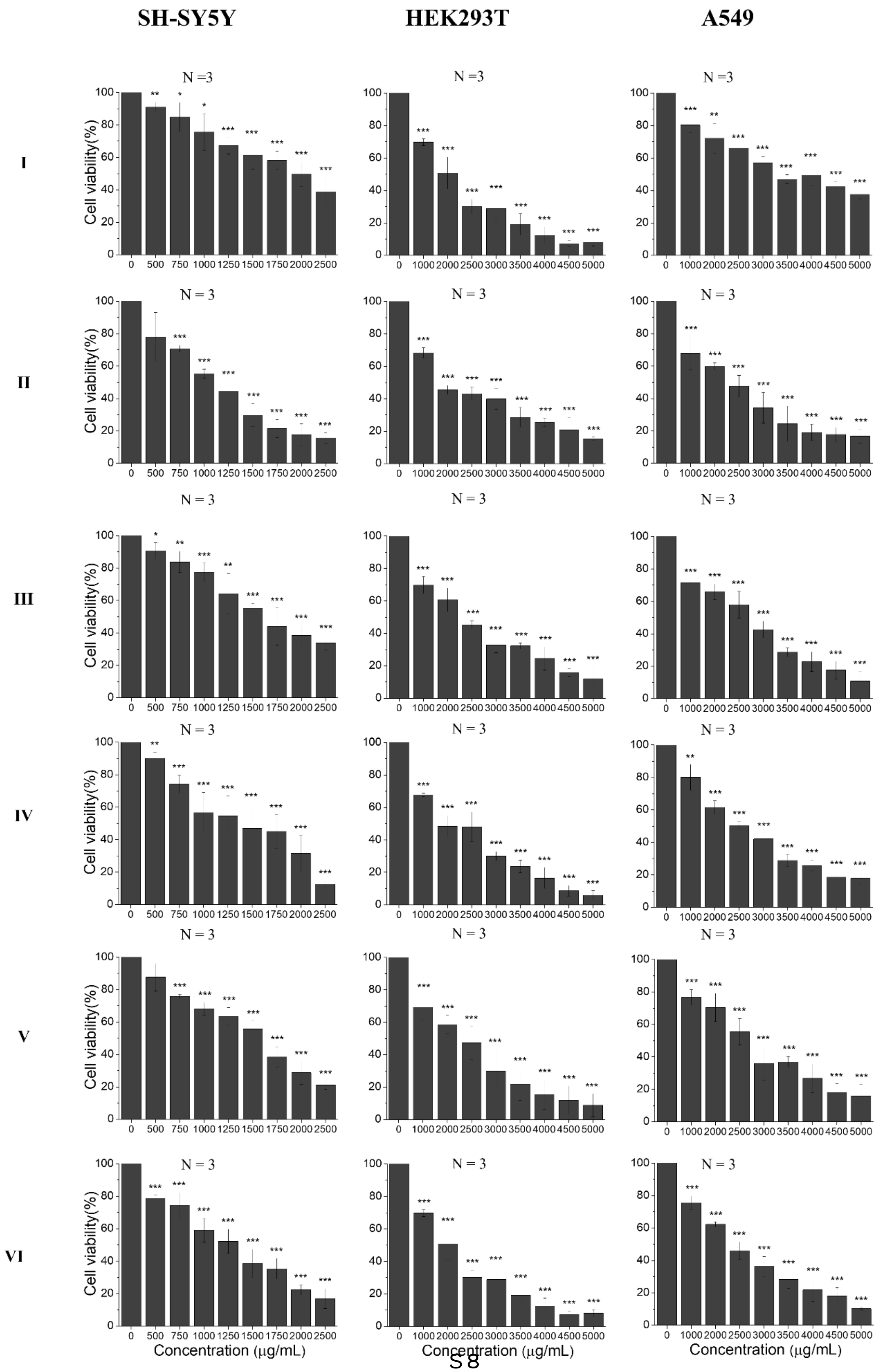
SH-SY5Y
HEK293T
A549
I
II
III
IV
V
VI
N =3
N = 3
Cell viability(%)
0 500 750 1000 1250 1500 1750 2000 2500
0 1000 2000 2500 3000 3500 4000 4500 5000
Concentration (μg/mL)

Figure S5 *Size-dependent WP CAE influence on living cells verified by MTT assay*. The dose response curve of *CAE* in SH-SY5Y, HEK293T, and A549 cell lines using the MTT assays. *, **, and *** represent significant differences (* = $p < 0.05$), (** = $p < 0.01$) and (*** = $p < 0.005$). The numbers (N) indicate the number of experiments conducted in each condition. I~VI indicates the size of cigarette aerosol obtained with MOUDI listed in Fig. 1.

(A) (B)

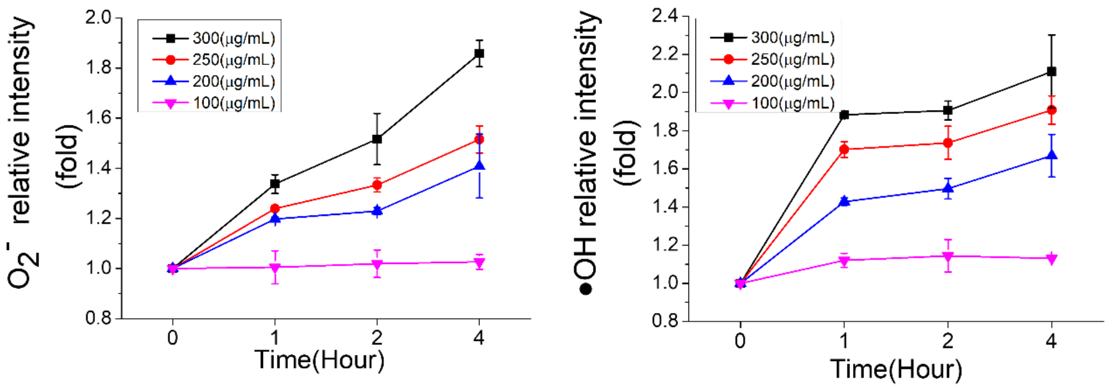

Figure S6 *The time-dependent and dose-dependent mitochondrial ROS in A549 cells treated with OP CAE (D_{PM} = 0.56 ~ 0.32 μm).* Detection of mitochondrial ROS (A). O_2^- and (B). •OH generation.

.

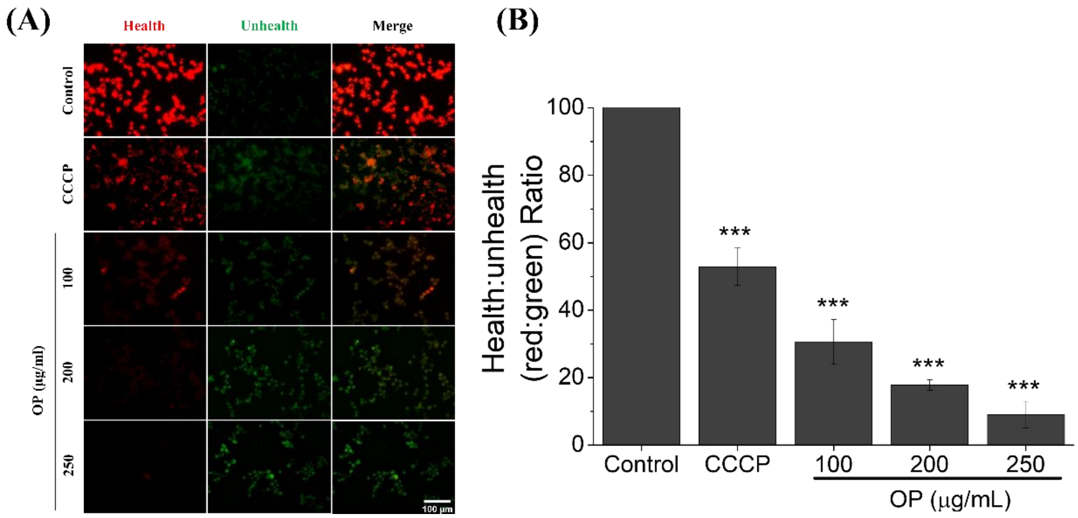

Figure S7 *The influence of OP CAE (D_{PM} = 0.56 ~ 0.32 μm) on mitochondrial potential verified by JC-1 assay.* (A). Confocal images of A549 cells stained with JC-1 after treatment of different concentrations of the OP CAE and 100μM CCCP (Potent mitochondrial oxidative phosphorylation uncoupler). Red fluorescence indicates healthy mitochondria, green fluorescence indicates unhealthy mitochondria. (B) The quantified fluorescence ratio was acquired with the microplate reader (Molecular Devices, SpectraMax iD3). *, **, and *** represent significant differences (* = $p < 0.05$), (** = $p < 0.01$) and (*** = $p < 0.005$).
